# The components of workplace violence against nurses from the perspective of women working in a hospital in Tehran: a qualitative study

**DOI:** 10.1186/s12905-021-01342-0

**Published:** 2021-05-19

**Authors:** Mitra Faghihi, Aliasghar Farshad, Maryam Biglari Abhari, Nammamali Azadi, Morteza Mansourian

**Affiliations:** 1 Health Education and Promotion, Department of Health Education & Promotion, School of Public Health, Iran University of Medical Sciences, Tehran, Iran; 2 Occupational Health Research Center, Iran University of Medical Sciences, Tehran, Iran; 3 Community Medicine Specialist Preventive Medicine and Public Health Research Center, Psychosocial Health Research Institute, Community and Family Medicine Department, School of Medicine, Iran University of Medical Sciences, Tehran, Iran; 4 Department of Biostatistics, School of Public Health, Iran University of Medical Sciences, Tehran, Iran; 5 Health Management and Economics Research Center, Iran University of Medical Sciences, Tehran, Iran

**Keywords:** Violence, Healthy workplace, Women's health, Nurse, Hospital

## Abstract

**Background:**

Based on the World Health Organization (WHO), workplace violence can affect events where employees are abused, attacked or threatened in their workplace, and it also has some consequences such as safety, welfare, and health. Like other types of violence, workplace violence and aggression are an increasing phenomenon. Moreover, workplace violence not only disrupts interpersonal and organizational relationships, but it also impairs the persons self-esteem and affects their physical and mental health and well-being. Thus, this study aimed to explain the components of workplace violence against nurses from the perspective of women working in a hospital in Tehran, which was conducted through the qualitative method and content analysis.

**Methods:**

In this study Purposive sampling included 21 female nurses who were working in different wards of the hospital. Also, female nurses were selected with maximum diversity in terms of work experience, age, and the wards they were working in. in this study the semi-structured interview was the main method of data collection. The interview transcriptions were extracted and then divided into meaningful units. For strengthening and confirming the results and accuracy of the research, the author used the data acceptability, credibility, accuracy, validity, believability, verifiability, reliability, and transferability.

**Results:**

During the data analysis process of this study, the first 15 classes with the same characteristics were put together and then divided into 6 classes. Afterwards, based on the common features at a more abstract level, they were converted into 2 themes. Based on the findings, violence against women in the workplace occurs at two levels, that namely interpersonal violence and organizational coercion.

**Conclusion:**

Therefore, it is necessary for managers to commit to lay the groundwork for reducing violence in the hospital, as well as barriers to report these cases especially the hospital managers and officials should create awareness on workplace among the staff, patient and visitors and must ensure stringent actions to prevent it.

## Background

According to the World Health Organization, violence is defined as "The intentional use of physical power or force, threatened or actual, against oneself, another person, or against a group or community, which either results in injury or has a high likelihood of resulting in death [1].

WHO described workplace violence as events where employees are abused, threatened or attacked at the workplace, the consequences of which affect their safety, well-being, and health. Moreover, in 2014, the American Nurses Association (ANA) added lateral violence, encompassing acts of violence among colleagues, bullying, hostility, power abuse, and sexual harassment into the definition of workplace violence [2].

However, women are among the vulnerable groups in any society due to physical, social, cultural, and traditional conditions. They can be exposed to a variety of problems such as human rights violations and gender-based violence. “Violence against women means any act of gender-based violence that results in, or is likely to result in, sexual, physical or psychological harm or suffering to women, including threats of such acts, coercion or arbitrary deprivation of liberty, whether occurring in public or in private life” [3].

Verbal, physical, and sexual insults are also frequent in workplaces [4]. Workplace violence does not only disrupts interpersonal and organizational relationships, but it also impairs people's selfesteem and affects their physical and mental health and well-being [5].

The incidence rate of workplace violence varies in different countries, ranging from 18.22% to 56% for physical violence, 63.8% to 89.58% for verbal abuse, and from 4.7% to 19.7% for sexual harassment [6]. A large multicenter study conducted on the incidence of workplace harassment by the European Foundation in 27 EU members in 2010 indicated that, among the approximate number of 48,000 people participating in the study, 5% of them had experienced some sorts of harassment at workplace in the last year [7].

In a systematic review stated that workers in the health, education, and public safety sectors are more prone to experience workplace violence. However, health care is a sector where violence is a major problem worldwide [8].

In health care, workplace violence is classified to both physical and mental violence. Obviously, physical violence is the most serious form of aggression, while psychological violence includes verbal abuse, threats, bullying, and sexual-racial harassment [6].

Nurses, due to their responsibilities, receive the highest rate of violent attacks in health care Centers [9] and are roughly 3 times more likely to be exposed to workplace violence than the employees working in other occupations [10]. In addition, they are more exposed to verbal, emotional, physical, and even sexual abuse [9, 11]. Based on the Occupational Safety and Health Administration (OSHA), 80% of serious violence in health centers occurs due to nurses' interactions with patients [2]. Also, Nurses' direct contact with patients and their families perhaps is one of the reasons why they are mostly abused. Nurses also spend more time with patients compared to other employees [4]. Such violence in the workplace can negatively affect the provision of medical services and create a hostile environment women and employees, and consequently affect the quality of service delivery. Moreover, if continued, it can face human resources management with several serious problems such as employee dissatisfaction, defect in the workflow, the reduced employee productivity, and absenteeism [10].

In addition, psychological distress can affect both men and women in different ways. Studies performed on post-traumatic stress disorder have shown that women are at a higher risk compared to men by about a ratio of 2 to 1, and estimates indicated that women are more exposed to anxiety about 1.4 to 1.8 times than men during their lifetime [1].

Harassment of women in the workplace is one of the examples of violence against them that demands the attention of society and politicians. Gender inequality and violence against women occur in a variety of workplaces and usually affect women with low-paid employment and low income levels [12].

In this regard, in a study, it was shown that there was an inverse relationship between quality of work life and the incidence of workplace violence [13]. Moreover, Sara Rizvi's study on female nurses in two hospitals in Pakistan showed that 73.1% of female nurses reported experiencing some forms of violence in the past 12 months, 53.4% of which were physical violence, 57.3% verbal violence, and 26.9% were sexual violence, and the main perpetrators of which were colleagues, patients, and patients' relatives [11]. In a study conducted by Behboodi Moghaddam et al., it was shown that the workplace harassment experience occurs when power relationships between women and men are unbalanced. In addition, women believe that their lifestyle affects harassment, which poses many problems, including low self-esteem and the reduced performance in the workplace [10]. Therefore, given the profound effects of this phenomenon on nurses in hospitals, this study aimed to investigate and explain the components of violence at workplace on female nurses working at a hospital in Tehran.

## Method

This paper is part of a qualitative study conducted in a hospital in Tehran. As a specialized general hospital in Tehran, this hospital provides medical services.

Qualitative research has provided important insights into the mental experience of violence and a greater understanding of the context and related meanings. However, independent quantitative and qualitative studies can separately make a significant contribution to understanding this complex phenomenon [14].

Therefore, this study was performed through the qualitative method and content analysis. Accordingly, content analysis is a method that provides a mental interpretation of the content of textual data and identifies the categories and classes or their related themes, by the use of a systematic cryptographic process. This type of analysis aims at achieving a well and concise description of the phenomenon that is under study as well as classifying the information obtained from this analysis into the themes or categories that describe the phenomenon [15]. The first author, who had good communication skills and interview experience, conducted the interviews. The interviewer was a PhD candidate in health education and health promotion who had excellent knowledge of workplace health. In this study, purposive sampling was used and female nurses working in different wards of the hospital were included in the study. In this regard, they were consciously asked to participate in this study and data were saturated after 21 interview sessions.

Regarding the maximum diversity in terms of age, work experience, and the ward where nurses work, 21 female nurses were enrolled in this study. The participants were completely willing and consented to participate in this study. The necessary arrangements were made with the ward nurse and supervisor or with staff and managers to participate in the research prior to the interview session, and permission was obtained from them. Data were collected at the interviewees’ workplace, in the ward where the interviewee worked, usually in the nurses' rest room without the presence of anyone other than the interviewer and interviewee. Moreover, they were given information on how the research and interview would be conducted, and they were excluded from the study if dissatisfied with participating in the research and interview. The nurses who were included in this study were assured that their information would remain confidential and also an informed consent was obtained to conduct interviews and recording conversations.

### Data collection method

The semi-structured interview was the main method of data collection. Interviews were started with the main question as "What aspects of workplace violence do you see here?" and then continued with some questions such as please explain more to give depth to the questions. The interview guide developed for this study is provided as additional Table 1.

**Table 1:** Interview guide

Interview guide			
1	Core question	Probe question	Field Notes
2	Explain about violence in the workplace		
3	How do you see violence in the hospital from different perspectives?	Explain more about each aspect?	
4	From whom do you experience violence?	Explain more about this	
5	What aspects may be insensibly considered as violence?	Explain more about this	
If we needed additional information, would we be allowed to contact you again?			

According to the hospital's work schedule, the duration of the interview was also adjusted with nurses, nurse heads or staff, ranging between 15 and 45 min. The interviewer met with the interviewees in person and interviewed them face to face. Everyone the interviewer intended to interview agreed with the interview. The interviews were recorded, and after each interview, the interviews were carefully transcribed and analyzed. After each interview, the interviewer provided it to the participants to comment on the accuracy of their statements. Before analyzing the interviews, the researcher read them for several times to get aware of their inner feelings and hidden meanings as well as gaining a general understanding of the interviews.

Afterward, the interview transcriptions were extracted and then divided into meaningful units, and important phrases related to the topic were extracted. In the initial coding, attempts were made to use the participants' words. Then, these codes were read many times and the similar codes dealing with a single subject were placed in one class and the codes were continuously controlled, and thus this coding was conducted in the second level or the same axial level. In the next step, the classes were compared and the similar classes were then merged to form a larger and more abstract class. MAXQDA was used to facilitate data analysis.

In the present study, for strengthening and confirming the results and research accuracy, the author used the data credibility, acceptability, validity, accuracy, believability, verifiability, reliability, and criteria for validation.

The participants were in constant conflict with the data and the data confirmation for the data acceptability and accuracy. During the interview, the researchers shared their impressions of the participants' speeches and opinions at appropriate times and made sure that their findings were correct.

Also, in order to increase the research validity, it has been directed under the supervision of professors with expertise in health promotion and experience in conducting qualitative research as well as the expert consultants in the field of education and promotion of health and workplace health. Besides performing in-depth semi-structured interviews to strengthen the data, secondary methods such as observing and recording field notes were used during the interview. For the transferability, to ensure the acceptance of the findings, the control of external observers was used in such a way that at each stage of information analysis to prevent abuse, data were provided to external counterparts for performing similar analysis. They then judged the findings and corrected their findings based on their points of views.

For the ethical considerations in this study, it was approved by the ethics committee in Iran University of Medical Sciences with the code of ethics No. IR.IUMS.REC.1398.201. Obtaining written informed consent, the right to withdraw from the study at any time were taken into account and maintaining privacy and confidentiality of participants' information as the ethical consideration of this research. In addition, to respect the rights of the participants, the arrangements were made for an interview. Written informed consent was obtained from all participants prior to taking part in this study. Also, the interviewees' permission was asked to record their voices and the research purpose, how it is conducted, and the data collection method were explained to them prior to conducting the interviews to ensure the confidentiality of the information and their participation. Moreover, before the interview, the consent of the participants in the project was obtained. The participants were then assured that the interview will remain confidential with the research team and in a safe place, and that they will have the right to discontinue the research at any time they wish to.

## Results

In this research, 21 female hospital staff nurses in Tehran aged between 24—40 years old and older with at least 2 years of work experience in the hospital, were included, 7 of whom were unmarried and 14 were married. Table 2 shows the participants' demographic information in the study. The two main categories of organizational and interpersonal violence are presented as central variables of the diagram. In the process of data analysis, the first 15 classes with the same characteristics were put together and then divided into 6 classes. The rest, based on common features at a more abstract level, they were turned into two themes. The findings indicates that violence against women in the workplace occurs at following two levels: interpersonal violence and organizational coercion. These two variables have all the characteristics of central variables. Table 3 shows Categories, Sub-Categories and Codes.

**Table 2:** Socio-demographic characteristics

	Socio-demographic variables	Percent (N)
Marital status	Married	66.6% (14)
	Single	33.3% (7)
Age in years	Less than 25	9.52% (2)
	25–30	19.04% (4)
	30–40	42.85% (9)
	Above 40	28.75% (6)
Work experience in years	10-Feb	30.09% (6)
	15-Oct	23.8%9 (5)
	15–20	19.04% (4)
	Above 20	19.04% (4)
Department	Men's surgery	9.52% (2)
	CCU	14.28% (3)
	Radiology	9.52%9 (2)
	Dialysis	14.28% (3)
	ICU	19.04% (4)
	Education	4.76% (1)
	Internal medicine	14.28% (3)
	Emergency	9.52% (2)
	Women's Surgery Department	4.76% (1)

**Table 3:** Categories, sub-categories and codes

Meaning unit	Code	Subcategory	Category
Unfortunately, there is so much workload and pressure that when we go home we take our tiredness home, and if your spouse says something, your child says something, we become agitated, and take whatever anger and violence we see there out on the poor ones …	Irrationally high workload	Organizational coercion	Organizational
"No one wants overwork of 50 or 100 h, but in our work overtime is mandatory, maybe I don't want to, but our job is mandatory and we all have to put in overtime work."	Forced shifts		
for example, they put me who work at CCU at the surgery ward, then put the person skilled in all the surgeries at the CCU, all of this will affect …	Compulsory placement in hospital wards		
"Every visiting patient is really stressing. When a child is hospitalized, I have to spend three hours trying to figure out why he is hospitalized? When I should discharge him? How is he going to afford the costs? These issues altogether start a stressful day or a harsh day … "	Workplace Stressors		
"Many of such things happen. The relative starts shouting and cursing, so why did it happen? Because there weren't enough beds in the emergency room	Lack of facilities		
"Nurses are always forced to consent, we are always forced to give consent, they never follow up, they were never our supporter, that if they were our supporter, it wouldn't be like this and no one would allow himself to disrespect the nurse …	Lack of managers' support during violence	vertical Violence	
"Some men officials can't understand women at all. Well, women have menstrual periods that hormonally agitates them. When a man is in charge, you can't go and say anything	Managers' lack of understanding of women's problems		
"Most of the times, people can easily harass a woman by bolding her relationship with the manager, and she is forced to neglect her job position to support her family, preferring not to make so much progress in her job position, but keep safe his family and the family foundation…"	Hidden violence against female nurses	Lateral or (horizontal) violence	Interpersonal
I believe that there is a hidden violence against women in the workplace, you know, in the Iranian administrative system, for example, if your abilities are a little greater than them and they see that you are getting ahead of them, they use that hidden violence. They use that force to show that you are a woman, and they can use this tool, it is very intangible though			
In two or three cases, I myself have seen that you, as a woman, are somehow vulnerable, though very intangible, especially they may start tongues a-wagging, and you may be somehow weakened, for example, it is so hidden, but it exists…			
we are not safe, we sit in the station, when we are two people, I have to wake up and my colleague can rest for an hour, I'm not safe at the station … and any moment the patient's relatives may come and argue, I try to take anything that might be dangerous out of hand, because maybe something hit you in the head any moment …"		Violence on the part of patients and companions	
"A statement that is said a lot, even insults that may be given in the emergency room are because their patient is not stable, and he is very upset and angry and sees his patient dying and is angry, and takes it out all on the defenseless nurses especially when his patient is critical, and it is very annoying	Patient and his relative's physical violence		
	Verbal violence of the patient or his relative		
	Patient ingratitude or patient companions		
"Sometimes because of the working conditions the personnel may do something, because we are dealing with patients, and my colleague may forget something, and in any case, in this crowd, for example, he was not paying attention, maybe because of that, we have disputes and conflicts… "	Verbal violence between staff	Among colleagues	
"I can't accept that the aggressiveness of nurse manager. I can't accept that the hospital head, the hospital manager, any of them have violence. They should advise in a calm environment, we have enough stress, it just worsens everything…"	Managers' verbal violence	managers	

Nurses are in direct contact with different segments of society and closely understand people's problems. Therefore, they have a serious responsibility. Various work-related factors such lack of manpower and equipment, long working hours, lifestyle, marital status have reduced health status of Iranian nurses, although work-related factors seem to play a more important role in this regard [16].

The bed-to-nurse ratio in Iranian hospitals is estimated to be %0.8. There are 20,000 nurses in Iran, while this figure should reach to 220,000 nurses, and consequently, this smaller number of nursing staff causes more pressure on employed nurses [17].

On the other hand, the difficulties of nursing work in Iran such as stress, job stress, and burnout, managerial model, role perception, less chance of organizational growth, have caused many nurses to leave the service, which in turn escalates the nursing problems in Iran [18]. On the other hand, Iranian women are considered the main pillar in household affairs. Therefore, considering their multiplicity of roles, the stress of working with patients and clients, problems related to night shifts and their less frequent attendance at family gatherings, will be more problematic. Thus, female nurses experience more challenges even than other working women in Iran [19].

## Organizational coercion (vertical violence)

Some cases such as irrationally high workload, forced shifts, forced placement in different wards of the hospital, low salaries, denial of benefits for over work, poor working environment, stressors in the workplace, and lack of facilities can be considered as organizational coercion (vertical violence) involving employees and nurses. Accordingly, many nurses refer to such cases as harassments that may not be taken seriously; however, these organizational problems can also affect the quality of life of these employees.

Irrationally high workload: Irrational workload for nurses can be known as one of the annoying factors in the workplace. Many factors can enhance the volume of nursing workload, which will also have psychological and physical effects on nurses in addition to compromising the quality of care and safety of patients. Many nurses attribute the shortage of human resources to forced shifts, and high workloads, which not only disrupt the work process and endanger their physical health, but it can also lead to several problems for them in the work and family environment due to some reasons such as interfering with the work and family life of these nurses, and thus "increasing the possibility of more work errors and creating the grounds for workplace violence":

"Very high workload, which is un-proportioned with the number of patients to nurses and is not standard; in addition to the reduced efficiency, an irrational increase in workload result in the other psychological problems …". (Participant No. 21).

"Unfortunately, there is so much workload and pressure that when we go home, we take our tiredness home, and if your spouse says something, your child says something else, we become agitated, and take whatever anger and violence we experience out there on the poor ones … " (Participant No. 20).

Forced shifts: Cases such as long and alternative shifts due to shortage of manpower, high work pressure, and the problems that may have resulted from these forced long work shifts may lead to increased physical and psychiatric health problems. In addition, these nurses suggest that these long and forced shifts may cause some problems in the family and normal life of these people. Cases statements are as follows:

"Our shits are circulating … and we must work about 100 h of overtime in one month. We really don't want to, but we don't have the strength, so we must come, and that's very annoying."(Participant No. 20).

"No one wants overwork of 50 or 100 h per month, but in our work, overtime is mandatory, maybe I don't want to, but our job is mandatory and we all have to put in overtime work."(Participant No15).

Compulsory placement in hospital wards: Another issue stated by nurses in different wards of this hospital was compulsory rotation placement of in wards at the discretion of the authorities.

Many of them acknowledged that when they become experts in a skill, changing wards without consulting them and compulsorily and with a top-down command can be annoying for them:

"I know how to work in this ward, when I see the patient from far away, I see his style, I know what I have to do and … but they say we have to work in all wards … for example, they put me at the surgery ward when I am working at the CCU, then put the person skilled in all the surgeries at the CCU, all of this will affect … they can ask our opinions … you can't send everyone to the CCU, right? But you can ask their opinions…"(Participant No. 03).

Workplace Stressors: Hospital workplace stressors can also be considered as one of the factors affecting nurses' perception of the harsh work environment. Although job stress exists in all work environments, because nurses are related to the health of other human beings, it brings more stress and more attention for nurses.

"Every visiting patient is really stressing. When a child is hospitalized, I have to spend three hours trying to figuring out why he/she is hospitalized? When I should discharge him/her? How is he/she going to afford the costs? These issues altogether start a stressful day or a harsh day …" (Participant No.02).

Lack of facilities: Lack of facilities is one of the issues that, also affects patients and their relatives in addition to affecting the way nurses provide services. Moreover, sometimes despite the constant efforts of nurses in the ward can raise various conditions leading to violence on the part of patients or their relatives.

"Many of such things happen. The relative starts shouting and cursing, so why does it happen?

Because there are no enough beds in the emergency room, if there are enough beds, then admitting the rest of the outpatients was possible through sufficient facilities, and we get no stress. " (Participant No. 14).

"When I easily say we don't have a device, it's wrecked, fix it as soon as possible, it's very hot here, they can fix a lot of things much easier, or prioritize it sooner, so that it can be fixed sooner." …; Not that one suffers so much that; for example, he has a lumbar disc and a neck injury and … then they finally begin to fix it so that it doesn't happen to them next, all of this put pressure on us and upset our nerves. (Participant No. 03).

Hidden violence against female nurses in the workplace: In the workplace, women may suffer hidden indirect violence, and this type of violence is considered so ugly that it is not easy to talk about. It can make them more vulnerable as well as affecting many of their abilities in a way that they try to avoid it because of their privacy as well as their family privacy.According to participants, many women may be accused of having an unusual relationship with a male manager due to a job promotion.

"Most of the times, people can easily harass a woman by bolding her relationship with her manager, and she is forced to neglect her job position to support her family, preferring not to make so much progress in her job position, to keep his family and the family foundation safe…" (Participant No. 17).

"I may find myself in a situation where for the sake of my job, I have to accept everything they ask me as a second person. It's a kind of violence in itself, and accepting it, does not end there, such psychological damage will be with me."(Participant No. 13).

On the other hand, some participants stated that women face false accusation considering their career promotion and advancement in various job categories and they are even slandered of having unusual relationships with male managers that are regarded by them as a form of violence.

In the Iranian administrative system, for example, if your abilities are a little greater than them and they see that you are getting ahead of them, they use that hidden violence. They use that force to show that you are a woman, and they can use this tool, it is very intangible though …. (Participant No. 03).

In two or three cases, I myself have seen that you, as a woman, are somehow vulnerable, though very intangible, especially they may start tongues a-wagging, and you may be somehow weakened, for example, it is so hidden, but it exists… (Participant No. 13).

Managers' lack of understanding of women's problems: Managers' lack of understanding on the problems in the workplace and nurses’ physiological differences, responsibilities, their role conflicts with men, and men's lack of understanding of their working conditions are other aspects that female nurses have expressed. This can disrupt their peace of mind at work and affect their mental health. In this regard, some nurses have stated the followings:

"Some male officials cannot understand women at all. Well, women have menstrual periods that hormonally agitates them. When a man is in charge, you can't go and say anything. For many times, I had a male supervisor and I had this problem, but he said you have to do it. I said, 'Sir, you have to provide me with an assistant, but he said no, you have to do it. If you were not feeling well, you had to take a leave and you wouldn't have to come. (Participant No. 16).

Lack of managers' support during violence against nurses: The managers' support for nurses can encourage them, and this support would also bring mutual respect between the patient and his relatives with the nurses, and the lack of support in times of crisis can lay the groundwork for more problems for nurses. According to many nurses, the followings were stated:

"Nurses are always forced to consent, we are always forced to give consent, they never follow up, and they were never our supporter, so if they were our supporter, it wouldn't be like this and no one would allow himself to disrespect a nurse …” (Participant No. 18).

"When something occurs and the patient screams and shouts, the right is given to the patient, they should listen to the staff, and should not judge one-sidedly, they shouldn't give the right to the patient in the first place …" (Participant No. 03).

## Violence on behalf of the patient and his relatives

Patient and his relative's physical violence: Patient and his relative's physical violence can occur due to some factors such as lack of facilities and force, resulting in the reduced quality of service or death. Also, the patient's sickness, and his relative's anxiety and stress with the patient's condition, put the nurse, physician, paramedic and hospital staff at risk. In this regard, the nurses stated that in these cases, the first person who is attacked and at risk of violence is a nurse; in addition, the hospital is located in the area, where due to the cultural conditions of the area and also having a contract with the prison around the city, criminals are sent to the hospital with soldiers, which could pave the way for violence. In following, a case stated that:

"This hospital made a contract with the prison, they bring the sick prisoners, then these prisoner patients may have done a thousand wrong things… we are not safe, we sit in the station, when we are two people, I have to wake up and my colleague can rest for an hour, I'm not safe at the station … and at any moment the patient's relatives may come and argue, I try to take anything that might be dangerous out of hand, because they maybe hit something in the head at any moment…"(Participant No. 20).

Verbal violence of the patient or his relative: Verbal violence of the patient or his relative, like the physical violence, may cause their anger for some reasons such as lack of facilities, poor health of the patient, the perception of nurse's poor performance or the demands of patients and relatives that are beyond the duties of nurses. Moreover, when something bad happens like death of a patient, the first one who is accused and abused is the nurse, and the patient's relatives who face the bad condition of their patient can target them for violence:

"A statement that is said a lot, even insults that may be given in the emergency room, are because their patient is not stable, and he is very upset and angry and sees his patient dying, so he is angry, and takes it out all on the defenseless nurses, especially when his patient is critical, and it is very annoying. (Participant No. 18).

Inter-personnel verbal violence: Verbal violence among the personnel can occur because of high work pressure, forgetfulness, and the resulting mistakes. Also, violence is mostly verbal and can be solved in most cases without others’ intervening.

"Sometimes because of the working conditions, the personnel may do something, because we are dealing with patients, and my colleague may forget something, and in any case, in this crowd; for example, he was not paying attention, maybe because of that, we have disputes and conflicts…")Participant No. 05(

Managers' verbal violence: Verbal violence of managers also is one of the aspects of violence expressed by female nurses for some reasons such as correctly doing things, the fast and correct process of working to show power to subordinates and nurses working in different departments.

Nurses believe that the violence on behalf of the patient or his relative is more tolerable, and they accept it more easily compared to the violence expressed by hospital managers and officials.

"I can't accept the aggressiveness of nurse manager. I can't accept that the hospital head, the hospital manager, any of them have violence. They should advise in a calm environment, we have enough stress, it just worsens everything…"(Participant No. 10).

## Discussion

Based on the findings of this study, nurses are exposed to all kinds of violence in their workplaces. Violence in workplaces such as hospitals, where employees interact with different people, both within their work system with their co-workers and managers, and outside the system with patients and their relatives takes on different dimensions. Although such violence sometimes clearly targets nurses and hospital staff, some of these cases covertly involve them, which cause a variety of physical, psychological, and social problems. However, such violence on behalf of patients and their relatives does not target employees and personnel, but in some cases the annoying behaviors of managers also causes trouble for them.

The data in this study showed that nurses in hospital systems may be subconsciously abused by the system and managers, irrationally high workload, high working shifts, and forced placement in different wards of the hospital. Despite the role conflict of women in the workplace, their low salaries and benefits compared to high work, stressors in the workplace, lack of facilities, poor work environment, and lack of understanding by managers about women's problems in the workplace were some of the issues that nurses expressed as violence. In a qualitative study conducted by Behboodi M et al. in Iran, physical harassment in the form of the forced labor and extra work was also classified as a component of workplace violence [10].In a study conducted by Strandmark et al. in Sweden entitled “health consequences of workplace bullying: experiences from the perspective of employees in the public service sector" it was also shown that financial harassment and high staff turnover may affect them; however, they may keep silent about it, and they would suffer some psychological damages [20]. Nurses' workload can also increase their work errors, which can affect the services to patients and treatment quality through excessive fatigue, thereby weakening the relationship between the nurse and the patient, and distort the nurse—doctor cooperation, which consequently put a lot of pressure on the nurse [21]. Regarding the fact that in nursing and hospital work, dealing with some stressors is unavoidable and factors such as high workload, staff shortage and excessive fatigue can worsen human error and cause violence against hospital staff and nurses, they are themselves stressors affecting nurses and leaving psychological effects for them [22].

The female nurses' high working pressure and the pressure of the multiple roles in the family and community are quite important factors that can leave negative psychological effects on them as well, if ignored by the organization. Many female nurses mentioned the role conflicts and a lack of work-life balance as factors affecting their working and family lives. In addition, ignoring the multiple roles that they have in the outdoor environment can be considered as a threat for them by the management. A study by Sharif Zadeh et al. indicated that nurses who lack a work–family life balance try to leave the organization. Moreover, the shortage of nurses and the resulting high workload, followed by an increased demand for services from patients and their families imposes a heavy workload on them, but when employees or nurses have high involvement and commitments in the family as well as their private life, they cannot respond to both sides and may leave the job [23].

A study by Alhani et al. indicated that as the amount of conflict increases on one side, the conflicts also increases on the other hand, which can affect the quality of life and work of individuals [24]. The lack of facilities in the hospital and the ward can cause violence for nurses and is known as a factor for provoking violence on behalf of patients or their relatives. In a study by Heydarikhayat N, one of the reasons for the occurrence of violence was the delayed patient care by the staff, lack of facilities, and low number of personnel [25].

One of the main themes of this study was interpersonal violence. Violence in the workplace can occur in different forms such as verbal, psychological, and even physical abuse, but due to the fear of being judged, being rejected, and lack of freedom of speech, its expression by people are limited and usually remain hidden.

In this study, cases such as making false statements regarding having work relationship with the male manager and career advancement that women may achieve are also classified as interpersonal violence. Participants stated that their colleagues accused of them of having unconventional relationships with male managers considering their career advancement and promotion, which is considered as a form of violence by colleagues and peers. Repetitive and abusive behaviors affect an employee's work performance, and occupational health [26]. Harassment in the workplace, especially if the gender-based one, is not only a personal issue but also an organizational concern [27] and the organization should identify these risks and inform employees and train them to deal with this type of harassment. It should also adopt policies and procedures that provide appropriate conditions for employees especially women to freely express such violence [28].

Hosseinabadi et al. reported that sexual violence is the rarest type of violence against nurses [29]. In a study by Honarvar et al., in 2019, it was shown that 68.4% of people are suffering from more than one type of violence and sexual abuse accounted for 10.8٪ of the cases. However, sexual harassment against healthcare workers is a problem for which there is a little information on how sexual harassment victims respond to this type of violence. Therefore, there is a need for conducting more studies using increasingly complex designs, rather than an exploratory model, which explain the complex relationships among harassment characteristics, institutional responses, and respondents over time [30]. In this study, no one directly reported sexual harassment, which seems to have various reasons. Accordingly, either this violence has not happened, which seems unlikely, or it has not been reported for various reasons. The most important reasons at first seem to be the cultural and modesty issues that people have, which prevent them from reporting, but this cannot be the only reason, so further investigations are needed for clarification.

One of the components of violence in the workplace in various articles was considered to be verbal violence. In this study, one of the components of violence in the hospital's workplace was by the patient and their relatives and that the nurses may have face most of it. Verbal violence, such as shouting, abusive language, and raising the voice were reported by interviewees expressed for some reasons such as speeding up work or paying attention by the patient or their relatives. Verbal threats and physical violence would have many side effects, including the reduced concentration at work, lack of attention to moral values, the increased error at work, even missing the working shifts in some cases, continuous absenteeism and disregarding the patient, the reduced job satisfaction, boredom with the work, loss of working days, and the increased resignation. In addition, these violence and verbal conflicts can be obstacles to the proper provision of services. In this regard, another effective perspective is the emphasis on the patient rights in line with the emphasis on the medical personnel rights, which will have a great effect on the work of nurses as well as the occurrence of violence[31]. In a study conducted by Muhammad W et al. in Jordan, the rate of violence was calculated as 91.4%, of which 95.3% were verbal [32]. A study by Kamchuchat et al. also showed that the most common form of violence is verbal violence by the patient and colleagues involving nurses [33]. Also, in a study conducted by Hemati E in Iran, it was shown that within the last year, all nurses were subjected to verbal violence by a relative or a patient, and the most common violence was against nurses, relatives, and patients. Most of the nurses took no action against them, and over half of the nurses stated that these incidents were not often reported by them because they thought that reporting or talking about these subjects is useless [34]. Also, a study by Rafati et al. showed that 72.5% of nurses experienced violence during their work period [35]. Moreover, Cheraghi et al. found that 74.1% of nurses faced violence at work, of which 64% were verbal abuse [36]. Also, a study by Babaei et al. showed that verbal violence is the most common type of violence faced by nurses and hospital staff in different wards, which has a rate of 66.2% [37]. In the study by Honarvar et al., verbal threats were 27.6% and verbal violence was 83.9%, and relatives, patients, and physicians played a role in expressing violence towards employees and nurses [2].

As a component of workplace violence, physical violence was another issue stated by the nurses.

One of the important reasons leading to physical violence in the hospital environment is the sickness or death of patients as well as expressing verbal and physical violence of their relatives towards hospital staff and nurses. In addition, due to the special environment of the hospital, because the criminals are hospitalized in this hospital, the security of this workplace will be endangered, especially for nurses. In a study by Babaei et al., physical violence was one of the aspects faced by nurses, and 4.9% reported this type of violence[37]. In the study by Juliana V. Magrin, physical violence was reported in 4.6% of female students in different forms such as slapping, pressing, hitting, kicking, suffocating, and threatening with a weapon. However, compared to the rate of emotional violence, physical violence was significantly less common [38].

In this study, physical violence was less emphasized than verbal violence, and the most reported violence by the patient or his relatives was verbal violence, which results in less physical conflict and violence and remains only verbal. A study by Gates et al. found that exposure to violent incidents was significantly associated with the reduced cognitive efficiency and the demand for support. The findings indicated that, while nurses express themselves, they can continue to function at a normal rate and are also able to provide safe and healthy care, but after a violent incident, they will experience more problems in cognitive and emotional terms [39].

Colleague violence, studied as horizontal violence in various literatures, was also described in this study by staff as a form of harassment that may be caused by some factors like high workloads as well as its problems such as improperly doing things or forgetting things, and a high stress level when working. A study by Hamblin LE et al. showed that power–work interdependence imbalance is a factor affecting violence and aggression, and working in stressful environments such as intensive care can be effective on causing this type of violence. If this violence becomes the culture of a workplace, it can also have a negative effect on employee retention[40]. A study by Taylor R found that nurses do not recognize behaviors associated with horizontal violence when observing and experiencing them. Accordingly, most of the interviewed nurses in the study did not identify their rape experiences as extreme violence, coercion or other terms in the literature or policies of violence in the workplace or code of conduct. Instead, they attributed it to some factors such as personality, work ethic, out of workplace life, or single events [41].

## Conclusion

The present study found that the expression of violence by patients and their relatives, whether verbally or physically, was accepted by nurses, but what most harassed them was the harassment and violence by their colleagues or managers. Patients and their relatives refer to the hospital in the worst possible physical and mental conditions and due to their pain and problems, they are not able to control their behaviors. In this regard, nurses and hospital staff consider such behaviors normal in critical situations despite the shortage of facilities and forces. Moreover, the idea that violence is an unavoidable aspect of nursing and hospital work, can prevent reports of violence, security measures, and lack of administrative commitment to address it. In order to reduce the level of violence in the workplace, especially in the hospital, it is necessary for managers to create opportunities for reducing violence in the hospital, as well as decreasing the barriers against reporting these cases. Also, managers and officials who are aware of violence in these work environments must make some efforts to prevent and reduce violence. Creating an environment in which the report of violence becomes a culture and presenting them to the management is performed in a clear and continuous manner to investigate and prevent it in the future, can be effective strategies on reducing violence cases.

## Data Availability

Data used in this study is analyzed and the data is available from the first author upon reasonable request.

## Abbreviations

WHO: World health organization
ANA: American nurses association
OSHA: Occupational safety and health administration
MAXQDA: Is a software program designed for computer-assisted qualitative and mixed methods data, text and multimedia analysis in academic, scientific, and business institutions
